# Trend of pregnancy outcomes in type 1 diabetes compared to control women: a register-based analysis in 1996-2018

**DOI:** 10.3389/fendo.2023.1232618

**Published:** 2023-07-12

**Authors:** Vince Fazekas-Pongor, Mark M. Svébis, David Major, Katalin Pártos, Norbert Dósa, Ágota Mészáros, Viktor J. Horváth, Beatrix A. Domján, László Zsirai, Adam G. Tabák

**Affiliations:** ^1^ Department of Public Health, Faculty of Medicine, Semmelweis University, Budapest, Hungary; ^2^ Department of Internal Medicine and Oncology, Faculty of Medicine, Semmelweis University, Budapest, Hungary; ^3^ Department of Gynecology and Family Planning, Istenhegyi Gene Diagnostic Center, Budapest, Hungary; ^4^ University College London (UCL) Brain Sciences, University College London, London, United Kingdom

**Keywords:** type 1 diabetes mellitus, pregnancy, trend, stillbirth, perinatal mortality, cesarean section, neonatal intensive care, APGAR score

## Abstract

**Introduction:**

In 1989, the St Vincent declaration aimed to approximate pregnancy outcomes of diabetes to that of healthy pregnancies. We aimed to compare frequency and trends of outcomes of pregnancies affected by type 1 diabetes and controls in 1996–2018.

**Methods:**

We used anonymized records of a mandatory nation-wide registry of all deliveries between gestational weeks 24 and 42 in Hungary. We included all singleton births (4,091 type 1 diabetes, 1,879,183 controls) between 1996 and 2018. We compared frequency and trends of pregnancy outcomes between type 1 diabetes and control pregnancies using hierarchical Poisson regression.

**Results:**

The frequency of stillbirth, perinatal mortality, large for gestational age, caesarean section, admission to neonatal intensive care unit (NICU), and low Appearance, Pulse, Grimace, Activity, and Respiration (APGAR) score was 2-4 times higher in type 1 diabetes compared to controls, while the risk of congenital malformations was increased by 51% and SGA was decreased by 42% (all p<0.05). These observations remained significant after adjustment for confounders except for low APGAR scores. We found decreasing rate ratios comparing cases and controls over time for caesarean sections, low APGAR scores (p<0.05), and for NICU admissions (p=0.052) in adjusted models. The difference between cases and controls became non-significant after 2009. No linear trends were observed for the other outcomes.

**Conclusions:**

Although we found that the rates of SGA, NICU care, and low APGAR score improved in pregnancies complicated by type 1 diabetes, the target of the St Vincent Declaration was only achieved for the occurrence of low APGAR scores.

## Introduction

Type 1 diabetes mellitus affects approximately 0.3% of pregnancies (1), and studies indicate that it is associated with a more frequent occurrence of several unwanted pregnancy outcomes (2, 3). Estimates show that adverse fetal outcomes, such as congenital malformations, perinatal mortality, preterm delivery, and large for gestational age infants (LGA), occur 2-5-fold more often among pregnancies affected by type 1 diabetes compared to healthy pregnancies (3). Furthermore, type 1 diabetes is also associated with other pregnancy-related adverse conditions, such as polyhydramnios, oligohydramnios (4), or more common occurrence of gestational hypertension and preeclampsia (5). As a result, pregnancies with type 1 diabetes require closer observation, and prospective mothers also undergo Caesarean section (C-section) more often with estimates going as high as 50% of pregnancies ending in surgery (3). In turn, infants born to mothers affected by type 1 diabetes require closer attention, as they often exhibit lower Appearance, Pulse, Grimace, Activity and Respiration Scores (APGAR) compared to their healthy counterparts (6) with up to 50% of infants being admitted to neonatal intensive care units (NICU) after delivery (3).

Since the incidence and prevalence of type 1 diabetes are increasing worldwide (7, 8), and this is accompanied by underlying changes in sociodemographic factors (for instance the continuous increase in maternal age and more frequent occurrence of births at earlier gestations) (9, 10) and the use of novel insulins, insulin delivery devices, and continuous glucose measurement devices, these changes may in turn also affect the trends of pregnancy outcomes in this subpopulation.

More than 30 years ago, diabetes specialists and healthcare policy makers approved the St Vincent declaration that set very ambitious targets and goals to substantially decrease disease burden and mortality related to diabetes (11). One area of these goals covered pregnancies complicated by diabetes: participants of the meeting unanimously agreed and pledged to approximate pregnancy outcomes of diabetes to that of healthy pregnancies (11). Since the declaration, however, very few studies have been conducted to actually test whether these goals have been met or to investigate the trajectories of outcomes of pregnancies complicated by type 1 diabetes both nationally and internationally. Thus, we aimed to investigate time trends of pregnancy outcomes in Hungary between 1996 and 2018 and to examine whether the targets outlined in the St Vincent declaration were achieved by comparing pregnancies affected by type 1 diabetes with pregnancies uncomplicated by type 1 diabetes using data of an anonymous registry of all deliveries in Hungary.

## Materials and methods

### Study design

The current report is based on the Tauffer registry that is a mandatory nation-wide registry of all deliveries in Hungary since the 1930s (4). Attending physicians are required to fill in a standardized, anonymous form after all deliveries between gestational weeks 24 and 42. Given that the database contains anonymous records, the identification of repeated deliveries by the same woman was not possible. The database is accessible in an electronic format for the years 1994 to 2018. For our present analysis, we included all singleton births between 1996 and 2018. The time restriction was used because underlying diseases are coded using the International Classification of Diseases (ICD) system, and its 10^th^ revision was introduced in Hungary in 1996 (12).

The legal basis of the anonymized registry is the 76/2004 ESzCsM decree (Decree on the Determination, Collection, Analysis of Health-related Unidentifiable data; Ministry of Health Social and Family Affairs, Hungary). Data presented in this report were made available through a data sharing agreement with the National Institute for Quality and Organizational Development in Healthcare and Medicines that confirmed that no ethical approval is required.

### Participants

**Figure 1:**
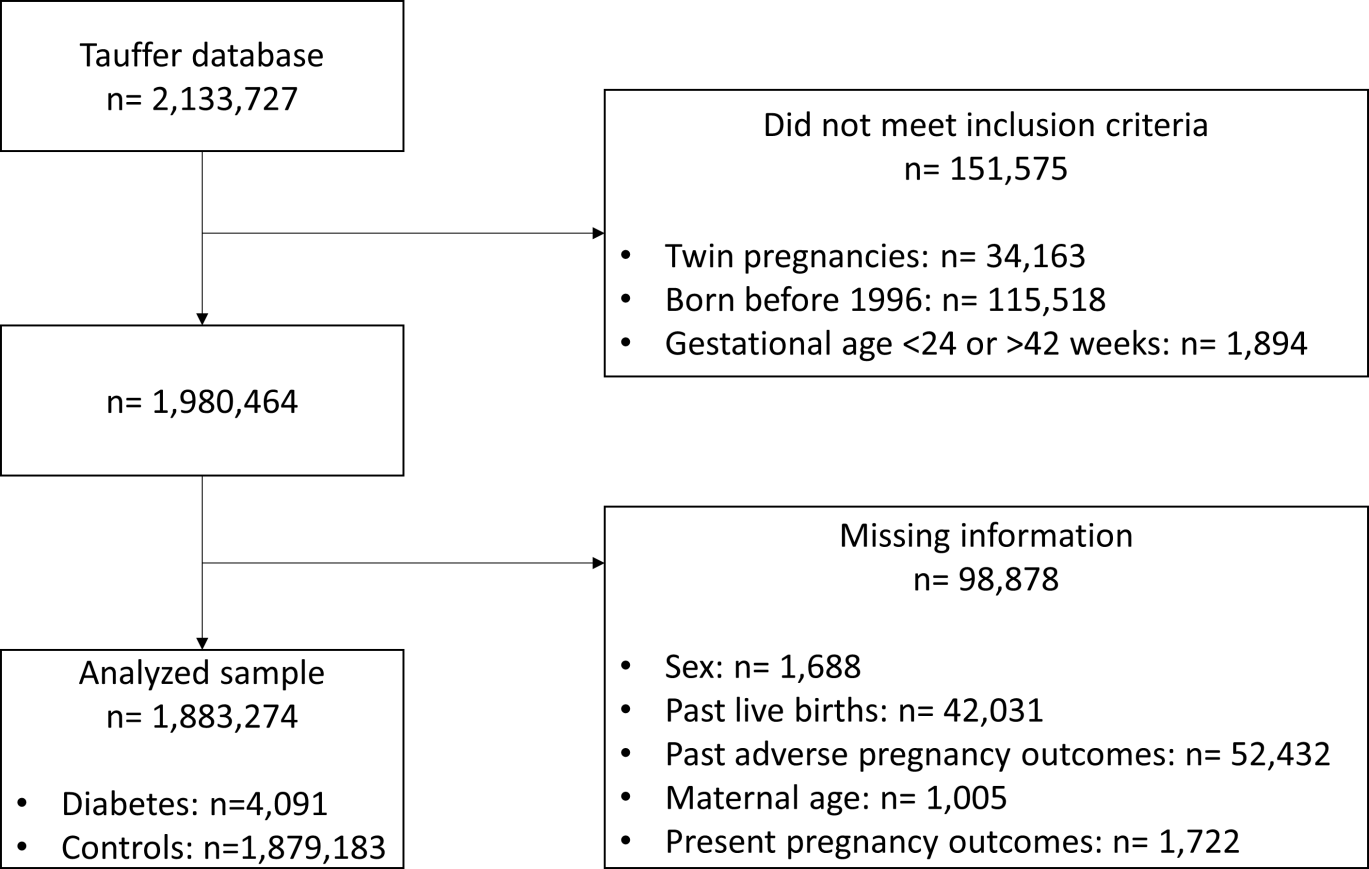
Flow chart of study participants.

Of the total of 2,133,727 births included in the Tauffer database between 1994 and 2018, we excluded 115,518 as these happened before 1996. We further excluded 34,143 twin pregnancies, 1,894 deliveries that had a gestational age recorded <24 or >42 weeks. Of the eligible 1,980,464 births, we excluded 98,878 cases due to missing information on outcomes or covariables. Thus, the final analytical sample consisted of 1,883,274 pregnancies (95.1% of those eligible, 4,091 affected by type 1 diabetes and 1,879,183 control pregnancies) (**Figure 1**).

### Variables

#### Exposure

The main exposure for the current analysis is the presence of type 1 diabetes mellitus. For all information related to index pregnancies, data were extracted from hospital discharge reports. Data on known diseases/pathologies before and during the investigated pregnancy was based on ICD-10 codes. Type 1 diabetes was coded either as a preexisting disease (E10*) or as a complication of the index pregnancy (O240, O243, and O249). Controls were all pregnancies without a mention of the previous ICD-10 codes (13).

#### Outcomes

Regarding outcomes, there were separate dedicated questions for stillbirth, perinatal mortality (any death between week 24 of pregnancy and day 7 postpartum), C-section (elective or emergency), and admission to the neonatal intensive care unit (NICU). Congenital malformations (Q*) were recorded as a separate entry. Using birthweight, fetal sex, and gestational age at delivery, we calculated percentiles that were translated to small for gestational age (SGA, birthweight <10^th^ percentile) and large for gestational age (LGA, birthweight >90^th^ percentile) (14). Similarly, the 5-minute APGAR scores that were recorded in the database were coded as low APGAR scores if they were below 7.

#### Covariates

Maternal age was calculated as the difference between date of delivery and date of birth. Gestational age at delivery was based in most cases on the first trimester crown-rump length or was calculated from the date of the last menstrual period. Newborn sex was based on the phenotype at birth. From the detailed account of past pregnancy histories, we extracted the following variables: past induced and spontaneous abortions, past stillbirths, and past livebirths (all coded as yes/no variables). Using the previous variables, we created a variable describing prior adverse pregnancy outcome if the history contained either a stillbirth or an abortion. Primary maternal hypertension (I10*, I11*, I12*, I15*) was recorded as a preexisting disease or complication of the index pregnancy (O10*, O11*, O16*).

### Statistical analysis

Given that the database contains anonymous records and thus the identification of repeated deliveries by the same woman cannot be identified, we assumed that outcomes could be correlated between repeated pregnancies and consequently the independence of observations may not hold true. Thus, for all estimations we used either bootstrapping or robust estimators.

#### Descriptive analysis

Descriptive statistics comparing pregnancies complicated by type 1 diabetes with control pregnancies was performed using independent sample t-tests for continuous variables and Chi-squared tests for categorical variables. Furthermore, we looked for temporal trends in descriptive variables within type 1 diabetes and control pregnancies. For this analysis, we decided to divide the 1996–2018 period into three periods (1996–2002, 2003–2010, 2011–2018). Heterogeneity and linear trends within the 3 periods were calculated with logistic regression for categorical variables and one-Way ANOVA for continuous variables.

#### Pregnancy outcome analysis

First, we compared pregnancy outcomes of pregnancies complicated by type 1 diabetes and controls using Poisson regression for the entire observation period. We calculated unadjusted and adjusted rate ratios (RR) with their respective 95% confidence intervals (95% CI). For the adjusted analysis, 2 different models were constructed. In *Model 1*, outcomes were adjusted for gestational age (linear, quadratic, and cubic terms), sex of the infant, and age of the mother (linear and quadratic terms). *Model 2* was further adjusted for the presence of prior adverse pregnancy outcome, prior livebirth, and pre-pregnancy hypertension. Then, we calculated rate ratios with respective 95% CIs using Poisson regression for each of the 3 time periods and investigated whether there was any heterogeneity or a linear trend over time. For this analysis, we also provide unadjusted and adjusted estimates as described previously. For those outcomes where a linear trend was likely (p<0.10) in the adjusted models (meaning that the relative difference either increased or decreased between cases and controls over time), we ran an additional Poisson regression model where calendar time was treated as a continuous variable. For this model, we added calendar time (linear, quadratic, and cubic terms) and an interaction between calendar time and presence of type 1 diabetes (linear term) as covariates in addition to variables in *Model 1*. We then calculated estimated marginal means for the proportion of the given pregnancy outcomes for each calendar year with respective 95% CIs and present them as band charts.

#### Sensitivity analysis

To overcome the potential multilevel structure of the data, we ran a sensitivity analysis exclusively on first pregnancies (thus here we excluded records with a livebirth or abortion in the medical history).

All analyses were conducted in IBM SPSS Statistics version 28.0.1 (IBM, Armonk, NY, US). Significance was set at p<0.05.

## Results

### Descriptive statistics

Altogether, 0.22% of all pregnancies were affected by type 1 diabetes. The proportion was stable in the first two periods at 0.20% but increased by 36% (OR: 1.36 95% CI: 1.26-1.46) in the last period to 0.26%.

Mothers affected by type 1 diabetes were 2.7 years older and gave birth more than a week earlier, although the sex distribution of the newborns were similar in cases and controls. Past pregnancy history revealed that mothers affected by type 1 diabetes were 14% (OR: 0.86, 95% CI: 0.81-0.92) less likely to be primi- or multiparas, but more likely to have had pregnancies ending with stillbirth (OR: 2.61, 95% CI: 2.04-3.34) or to have undergone either spontaneous (OR: 1.75, 95% CI: 1.62-1.88) or induced abortions (OR: 1.10, 95% CI: 1.02-1.19). Primary hypertension occurred more than 4 times more often among pregnancies affected by type 1 diabetes compared to control pregnancies (OR: 4.81, 95% CI: 4.00-5.78) (**Table 1** **).**

**Table 1:** Baseline characteristics of pregnant women stratified by type 1 diabetes status in 1996–2018.

	Type 1 Diabetes	Control	p-value*
	n=4,091	n=1,879,183	
Maternal age, years (SD)	31.5 (5.41)	28.8 (5.75)	<0.001
Gestational age at delivery, weeks (SD)	37.6 (2.30)	38.8 (1.92)	<0.001
Infant sex, n (%)			0.425
male	2,145 (52.4)	973,576 (51.8)	
female	1,946 (47.6)	905,607 (48.2)	
Past live births			<0.001
None	2,114 (51.7)	901,953 (48.0)	
≥1	1977 (48.3)	977,230 (52.0)	
Past stillbirths			<0.001
None	4026 (98.4)	1,867,637 (99.4)	
≥1	65 (1.60)	11,546 (0.60)	
Past induced abortions			<0.001
None	3,185 (77.9)	1,616,103 (86.0)	
≥1	906 (22.1)	263,080 (14.0)	
Past spontaneous abortions			0.017
None	3,342 (81.7)	1,561,431 (83.1)	
≥1	749 (18.3)	317,752 (16.9)	
Any previous adverse pregnancy outcome			<0.001
None	2,597 (63.5)	1,355,182 (72.1)	
≥1	1,494 (36.5)	524,001 (27.9)	
Primary hypertension			<0.001
No	3,973 (97.1)	1,867,638 (99.4)	
Yes	118 (2.90)	11,545 (0.60)	

APGAR Score, Appearance, Pulse, Grimace, Activity and Respiration Score.

Adverse pregnancy outcome: either stillbirth or abortion in the medical history.

*p-values were calculated with chi-squared test for categorical variables and independent sample t-test for continuous.

When investigating trends in descriptive characteristics in women with type 1 diabetes and controls over the three time periods, we found significant linear increases or decreases for all descriptive characteristics except for infant sex in controls and infant sex, primi- or multiparity, and history of induced abortions for type 1 diabetes. Maternal age increased by over 3 years, while the occurrence of primary hypertension more than doubled in both groups. The frequency of primi- or multiparity increased and past induced abortion decreased only in controls, while past stillbirth decreased, and history of spontaneous abortion increased in both cases and controls (**Table 2**).

**Table 2:** Temporal trends in baseline characteristics of pregnant women by type 1 diabetes status over 3 time periods (1996–2002, 2003–2010, 2011–2018).

	Type 1 Diabetes (n=4,091)				Controls (n=1,879,183)			
	1996–2002	2003–2010	2011–2018	P for trend*	1996–2002	2003–2010	2011–2018	P for trend*
n (%)	859 (21.0)	1407 (34.4)	1825 (44.6)	–	476,635 (25.4)	715484 (38.1)	687064 (36.5)	–
Maternal age, years (SD)	29.5 (5.52)	31.0 (5.00)	32.9 (5.32)	<0.001	26.9 (5.26)	28.8 (5.40)	30.1 (6.06)	<0.001
Gestational age, weeks (SD)	37.8 (2.19)	37.6 (2.36)	37.5 (2.30)	0.002	38.9 (2.00)	38.8 (1.92)	38.7 (1.86)	<0.001
Infant sex, n (%)				0.072				0.793
male	476 (55.4)	731 (52.0)	938 (51.4)		247,165 (51.9)	345,118 (48.2)	331,019 (48.2)	
female	383 (44.6)	676 (48.0)	887 (48.6)		229,470 (48.1)	370,366 (51.8)	356,045 (51.8)	
Past live births				0.064				<0.001
None	415 (48.3)	739 (52.5)	960 (52.6)		218,299 (45.8)	342,060 (47.8)	341,594 (49.7)	
≥1	444 (51.7)	668 (47.5)	865 (47.4)		258,336 (54.2)	373,424 (52.2)	345,470 (50.3)	
Past stillbirths				0.033				<0.001
None	837 (97.4)	1,388 (98.6)	1,801 (98.7)		463,201 (99.3)	711,200 (99.4)	683,236 (99.4)	
≥1	22 (2.60)	19 (1.40)	24 (1.30)		3,434 (0.70)	4284 (0.60)	3,828 (0.60)	
Past induced abortion				0.064				<0.001
None	682 (79.4)	1,146 (81.4)	1,514 (83.0)		383,869 (80.5)	591,969 (82.7)	585,593 (85.2)	
≥1	177 (20.6)	261 (18.6)	311 (17.0)		92,766 (19.5)	123,515 (17.3)	101,471 (14.8)	
Past spontaneous abortion				0.025				<0.001
None	673 (78.3)	1,125 (80.0)	1,387 (76.0)		411,767 (86.4)	616,651 (86.2)	587,685 (85.5)	
≥1	186 (21.7)	282 (20.0)	438 (24.0)		64,868 (13.6)	98,833 (13.8)	99,379 (14.5)	
Any previous adverse pregnancy outcome				0.456				<0.001
None	523 (60.9)	920 (65.4)	1,154 (63.2)		333,588 (70.0)	514,640 (71.9)	506,954 (73.8)	
≥1	336 (39.1)	487 (34.6)	671 (36.8)		143,047 (30.0)	200,844 (28.1)	180,110 (26.2)	
Primary hypertension				<0.001				<0.001
No	847 (98.6)	1,375 (97.7)	1,751 (95.9)		474,859 (99.6)	712,254 (97.7)	680,525 (99.0)	
Yes	12 (1.40)	32 (2.30)	74 (4.10)		1,776 (0.40)	3,230 (0.50)	6,539 (1.00)	

APGAR Score, Appearance, Pulse, Grimace, Activity and Respiration Score.

Adverse pregnancy outcome: either stillbirth or abortion in the medical history.

*Linear trends were examined with logistic regression for categorical variables and ANOVA for continuous variables.

### Pregnancy outcomes

According to our unadjusted analyses, the frequency of all outcomes was significantly different in cases and controls. The frequency of stillbirth, perinatal mortality, LGA, C-section, admission to NICU, and low APGAR score was 2-4 times higher in type 1 diabetes pregnancies compared to controls, while the risk of congenital malformations was increased by only 51% and the risk of SGA was decreased by 42%. These observations remained significant after adjustment for potential confounders across all outcomes except for low APGAR Scores, which showed similar risks in cases and controls in the full model. More marked attenuation was found for stillbirth, perinatal mortality, NICU treatment, and APGAR score (>50% reduction in model betas), while the change was smaller for the other outcomes (**Table 3**).

**Table 3:** Rate ratios for different pregnancy outcomes based on Poisson regression with hierarchical adjustment for potential confounders in singleton pregnancies affected by type 1 diabetes compared to controls in 1996–2018.

	Type 1 diabetes	Controls	Unadjusted	Model 1†	Model 2††
	n=4,091	n=1,879,183			
	n (%)	n(%)	RR (95% CI)	RR (95% CI)	RR (95% CI)
			2000–2018		
Stillbirth	73 (1.80)	9105 (0.50)	3.68 (2.93–4.63)	1.82 (1.46–2.27)	1.82 (1.46–2.26)
Perinatal mortality	86 (2.10)	12,812 (0.70)	3.09 (2.50–3.81)	1.61 (1.32–1.96)	1.88 (1.53–2.32)
Small for gestational age	276 (6.70)	216,361 (11.5)	0.58 (0.52-0.65)	0.66 (0.59-0.74)	0.65 (0.58-0.73)
Large for gestational age	1,230 (30.1)	209,258 (11.1)	2.71 (2.58-2.85)	2.39 (2.27-2.52)	2.45 (2.33-2.58)
Caesarean section	2,454 (60.0)	544,184 (29.0)	2.07 (2.02-2.13)	1.80 (1.76-1.85)	1.70 (1.65-1.74)
NICU	862 (21.1)	107,201 (5.7)	3.70 (3.48-3.92)	2.12 (1.98-2.26)	2.05 (1.93-2.19)
Congenital malformations	112 (2.70)	34,159 (1.80)	1.51 (1.26-1.81)	1.44 (1.20- 1.72)	1.42 (1.18-1.70)
APGAR Score ≤6	58 (1.40)	19,234 (1.00)	2.08 (1.59-2.72)	1.36 (1.04-1.78)	1.36 (1.02-1.80)

APGAR Score: Appearance, Pulse, Grimace, Activity and Respiration Score; 95% CI, 95% Confidence Interval; NICU, Neonatal Intensive Care Unit; RR, Rate Ratio.

†Model 1 was adjusted for gestational age, maternal age, and sex of infant.

††Model 2 was adjusted for covariates of Model 1 and presence of prior adverse pregnancy outcome, prior livebirth, and pre-pregnancy hypertension.

When we looked for time trends over the 3 time periods, we found significant linear trends for all outcomes in controls: decreasing trends for stillbirth, perinatal mortality, SGA, and NICU treatment, while increasing for LGA, C-section, congenital malformations, and low APGAR score. As for pregnancies affected by type 1 diabetes, decreasing trends were observed for low APGAR score and increasing trend for C-section, and non-significant (p<0.1) increase in LGA and decrease in SGA, NICU care, and low APGAR score (**Tables 4**, **5**).

**Table 4:** Rate ratios for different pregnancy outcomes based on Poisson regression with hierarchical adjustment for potential confounders in singleton pregnancies affected by type 1 diabetes compared to controls over 3 time periods (1996–2002, 2003–2010, 2011–2018).

		Type 1 diabetes	Controls	Unadjusted	Model 1†	Model 2††
		n=4,091	n=1,879,183			
	Time period	n (%)	n (%)	RR (95% CI)	RR (95% CI)	RR (95% CI)
n	1996–2002	859 (21.0)	476635 (25.4)	–	–	–
	2003–2010	1407 (34.4)	715484 (38.1)	–	–	–
	2011–2018	1825 (44.6)	687064 (36.5)	–	–	–
Stillbirth	1996–2002	15 (1.70)	2,650 (0.60)	3.14 (1.90–5.19)*	1.81 (1.11–2.95)*	1.78 (1.09–2.92)*
	2003–2010	32 (2.30)	3430 (0.50)	4.74 (3.36–6.69)*	2.24 (1.63–3.09)*	2.24 (1.63–3.07)*
	2011–2018	26 (1.40)	3,025 (0.40)	3.24 (2.21–4.75)*	1.53 (1.05–2.23)*	1.52 (1.04–2.21)*
	P for heterogeneity**	0.199	<0.001	0.244	0.306	0.294
	P for trend**	0.342	<0.001	0.867	0.406	0.414
Perinatal mortality	1996–2002	17 (2.00)	4,185 (0.90)	2.25 (1.41–3.61)*	1.45 (0.91–2.31)	1.93 (1.21–3.09)*
	2003–2010	38 (2.70)	4,779 (0.70)	4.05 (2.96–5.55)*	1.97 (1.50–2.58)*	2.21 (1.64–2.97)*
	2011–2018	31 (1.70)	3,848 (0.60)	3.03 (2.14–4.31)*	1.48 (1.05–2.08)*	1.70 (1.18–2.44)*
	P for heterogeneity**	0.142	<0.001	0.114	0.337	0.541
	P for trend**	0.376	<0.001	0.401	0.865	0.534
Small for gestational age	1996–2002	67 (7.80)	62,981 (13.2)	0.59 (0.47–0.74)*	0.67 (0.53–0.84)*	0.66 (0.52–0.83)*
	2003–2010	101 (7.20)	79,242 (11.1)	0.65 (0.54–0.78)*	0.72 (0.60–0.87)*	0.71 (0.59–0.86)*
	2011–2018	108 (5.90)	74,138 (10.8)	0.55 (0.46–0.66)*	0.61 (0.51–0.73)*	0.60 (0.50–0.72)*
	P for heterogeneity**	0.142	<0.001	0.455	0.456	0.421
	P for trend**	0.052	<0.001	0.546	0.465	0.429
Large for gestational age	1996–2002	224 (26.1)	49,177 (10.3)	2.53 (2.26–2.83)*	2.22 (1.98–2.49)*	2.29 (2.05–2.57)*
	2003–2010	449 (31.9)	84,155 (11.8)	2.71 (2.51–2.93)*	2.44 (2.26–2.64)*	2.52 (2.33–2.72)*
	2011–2018	557 (30.5)	75,926 (11.1)	2.76 (2.58–2.96)*	2.46 (2.30–2.64)*	2.53 (2.36–2.72)*
	P for heterogeneity**	0.011	<0.001	0.419	0.297	0.311
	P for trend**	0.061	<0.001	0.299	0.212	0.236

95% CI, 95% Confidence Interval; RR, Rate Ratio.

*p<0.05.

**p-values for heterogeneity and linear trend of rate ratios were computed with Poisson regression.

†Model 1 was adjusted for gestational age, maternal age, and sex of infant.

††Model 2 was adjusted for covariates of Model 1 and presence of prior adverse pregnancy outcome, prior livebirth, and pre-pregnancy hypertension.

**Table 5:** Rate ratios for different pregnancy outcomes based on Poisson regression with hierarchical adjustment for potential confounders in singleton pregnancies affected by type 1 diabetes compared to controls over 3 time periods (1996–2002, 2003–2010, 2011–2018).

		Type 1 diabetes	Controls	Unadjusted	Model 1†	Model 2††
		n=4,091	n=1,879,183			
	Time period	n (%)	n (%)	RR (95% CI)	RR (95% CI)	RR (95% CI)
n	1996–2002	859 (21.0)	476635 (25.4)	–	–	–
	2003–2010	1407 (34.4)	715484 (38.1)	–	–	–
	2011–2018	1825 (44.6)	687064 (36.5)	–	–	–
Caesar section	1996–2002	427 (49.7)	92,751 (19.5)	2.55 (2.39–2.73)*	2.29 (2.13–2.45)*	2.18 (2.04–2.34)*
	2003–2010	810 (57.6)	200,723 (28.1)	2.05 (1.96–2.15)*	1.86 (1.77–1.94)*	1.76 (1.68–1.84)*
	2011–2018	1,217 (66.7)	250,710 (36.5)	1.83 (1.77–1.89)*	1.63 (1.57–1.68)*	1.54 (1.49–1.59)*
	P for heterogeneity**	<0.001	<0.001	<0.001	<0.001	<0.001
	P for trend**	<0.001	<0.001	<0.001	<0.001	<0.001
NICU	1996–2002	179 (20.9)	28,735 (6.00)	3.46 (3.04–3.94)*	2.07 (1.79–2.41)*	2.01 (1.73–2.34)*
	2003–2010	335 (23.8)	39,195 (5.50)	4.35 (3.96–4.78)*	2.47 (2.22–2.75)*	2.39 (2.14–2.66)*
	2011–2018	348 (19.1)	39,271 (5.70)	3.34 (3.03–3.67)*	1.88 (1.71–2.07)*	1.83 (1.66–2.01)*
	P for heterogeneity**	0.005	<0.001	<0.001	<0.001	0.001
	P for trend**	0.085	<0.001	0.245	0.085	0.091
Congenital malformations	1996–2002	25 (2.90)	8,255 (1.70)	1.68 (1.14–2.47)*	1.60 (1.09–2.36)*	1.59 (1.08–2.34)*
	2003–2010	42 (3.00)	13,418 (1.90)	1.60 (1.18–2.15)*	1.52 (1.13–2.05)*	1.51 (1.12–2.03)*
	2011–2018	45 (2.50)	12,486 (1.80)	1.36 (1.02–1.81)*	1.30 (0.97–1.73)*	1.28 (0.96–1.71)
	P for heterogeneity**	0.630	<0.001	0.622	0.639	0.610
	P for trend**	0.426	0.004	0.327	0.340	0.316
APGAR Score ≤6	1996–2002	19 (2.20)	4,046 (0.80)	2.61 (1.67–4.07)*	2.00 (1.29–3.12)*	1.97 (1.27–3.06)*
	2003–2010	20 (1.40)	4,166 (0.60)	2.45 (1.58–3.78)*	1.47 (0.96–2.26)	1.44 (0.94–2.22)
	2011–2018	19 (1.00)	11,022 (1.60)	1.56 (0.98–2.47)	0.95 (0.60–1.50)	0.93 (0.59–1.48)
	P for heterogeneity**	0.117	<0.001	0.232	0.070	0.070
	P for trend**	0.041	<0.001	0.118	0.028	0.028

APGAR Score: Appearance, Pulse, Grimace, Activity and Respiration Score; 95% CI, 95% Confidence Interval; NICU, Neonatal Intensive Care Unit; RR, Rate Ratio.

*p<0.05.

**p-values for heterogeneity and linear trend of rate ratios were computed with Poisson regression.

†Model 1 was adjusted for gestational age, maternal age, and sex of infant.

††Model 2 was adjusted for covariates of Model 1 and presence of prior adverse pregnancy outcome, prior livebirth, and pre-pregnancy hypertension.

**Figure 2:**
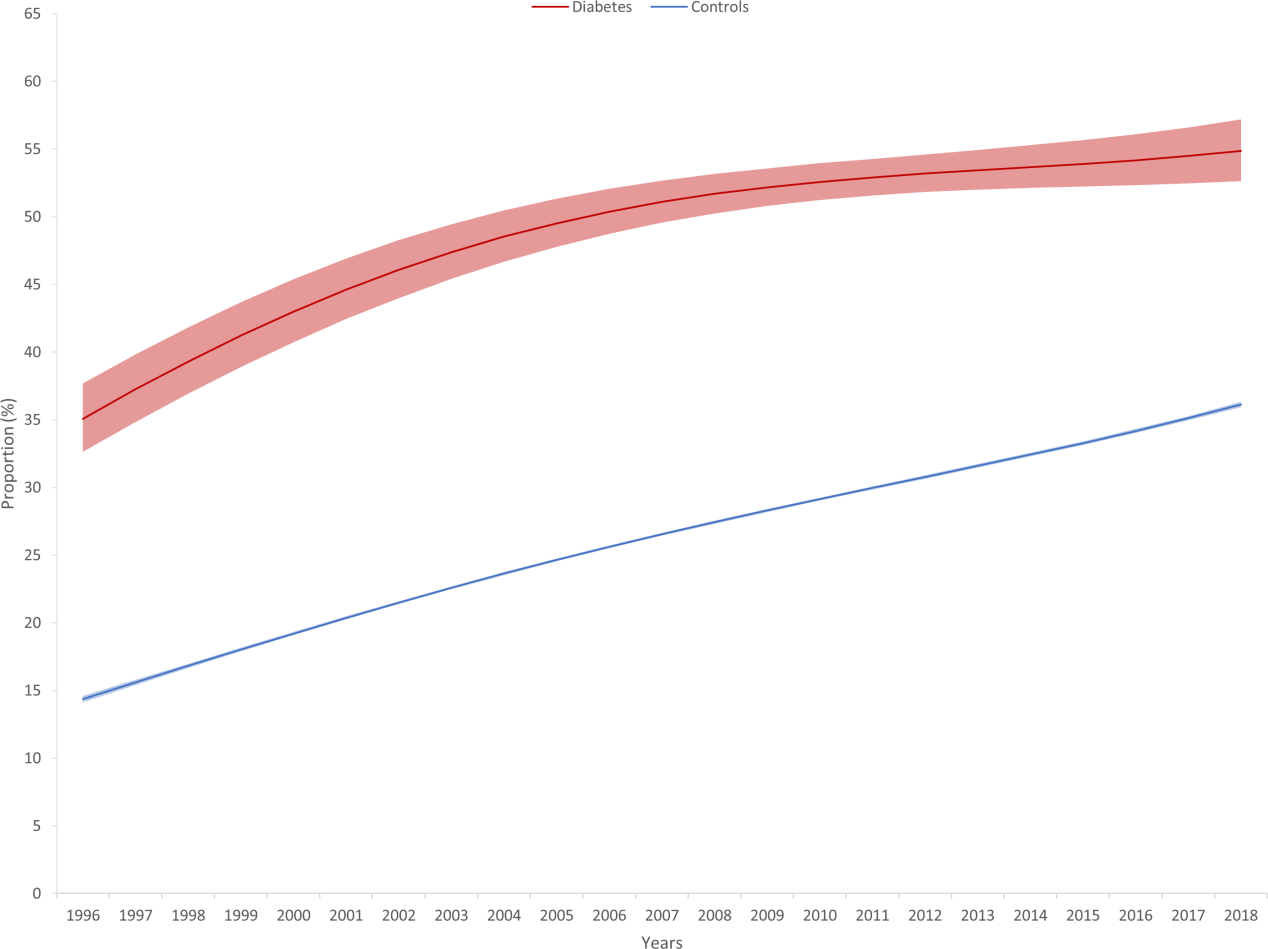
Estimated frequency of Caesarian sections (with 95% confidence intervals) based on Poisson regression with adjustment for gestational age at delivery, maternal age, and infant sex in singleton pregnancies affected by type 1 diabetes and controls from 1996 to 2018.

**Figure 3:**
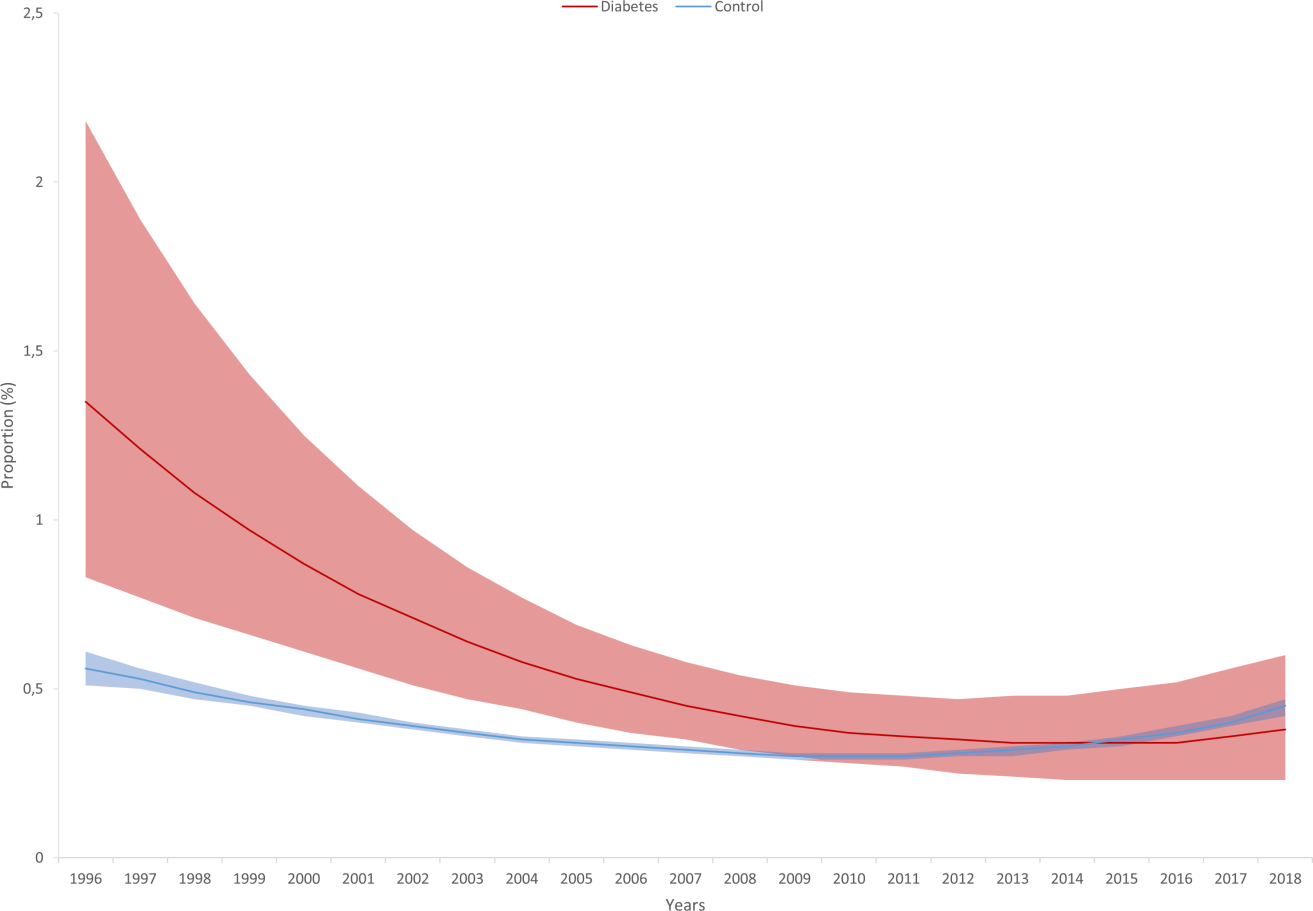
Estimated frequency of low APGAR (Appearance, Pulse, Grimace, Activity and Respiration) score (with 95% confidence intervals) based on Poisson regression with adjustment for gestational age at delivery, maternal age, and infant sex in singleton pregnancies affected by type 1 diabetes and controls from 1996 to 2018.

**Figure 4:**
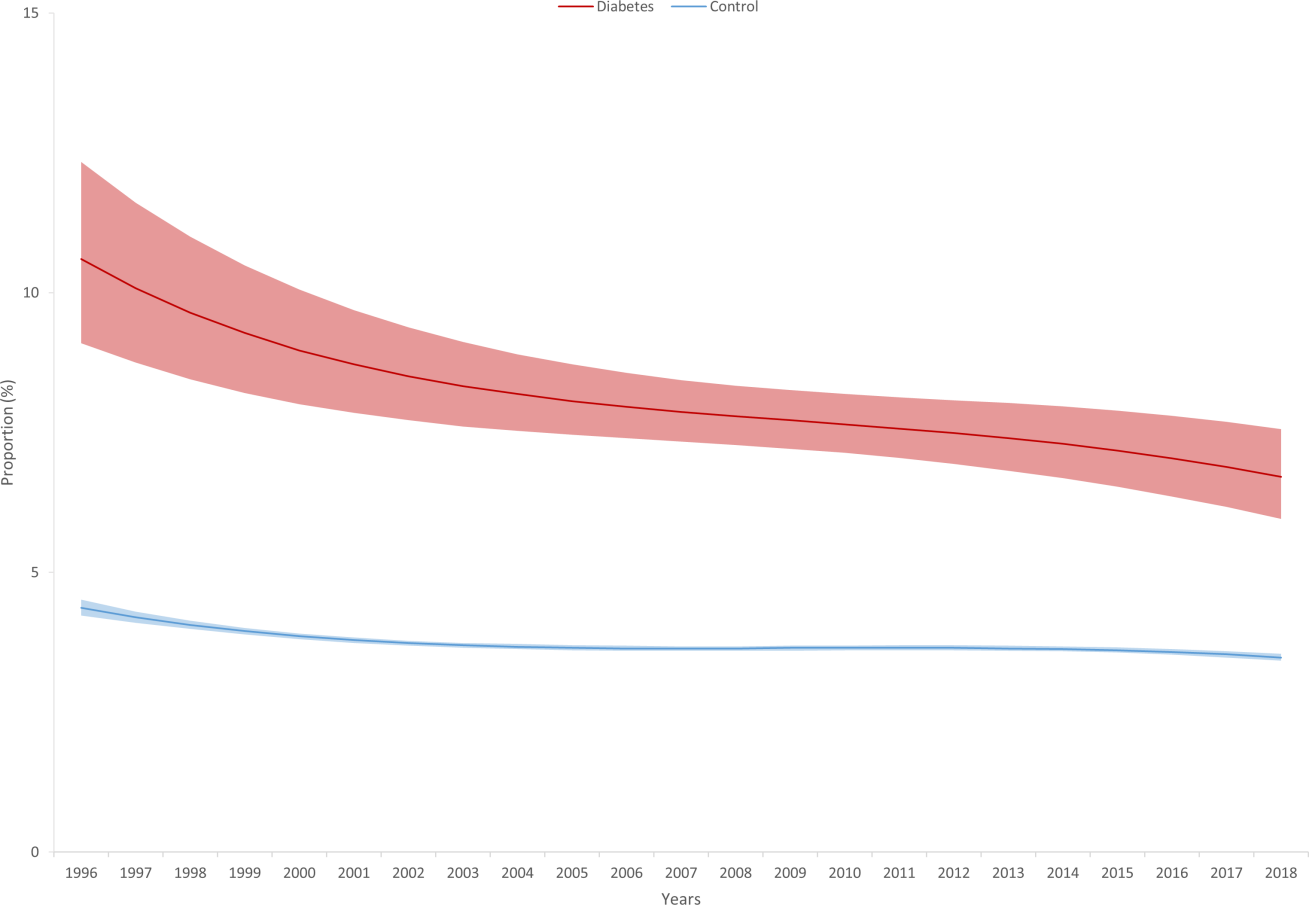
Estimated frequency of admission to neonatal infant care unit (with 95% confidence intervals) based on Poisson regression with adjustment for gestational age at delivery, maternal age, and infant sex in singleton pregnancies affected by type 1 diabetes and controls from 1996 to 2018.

When investigating trends in the RRs comparing cases and controls, we found a significant decrease for only C-sections in unadjusted models showing that the difference between cases and controls decreased over the observation period although the risk remained elevated in cases compared to controls even at the end of the observation period. This observation still holds in the adjusted models. Furthermore, we found that the RRs significantly decreased for low APGAR scores in the adjusted models and became non-significant in the 3^rd^ period. The figure showing the estimated marginal means shows that the gap decreased over the observation period and the confidence intervals were overlapping after 2009 (**Tables 4**, **5**; **Figures 2**, **3**). Although the gap decreased for admissions to NICU, the time by diabetes interaction term remained non-significant (p=0-052), indicating a possible power issue (**Figure 4**).

### Sensitivity analysis

Our sensitivity analysis on primiparas largely confirmed our main analysis on the point estimates, although, given the lower statistical power, some of the differences became non-significant (**Supplement Tables 1, 2**).

## Discussion

Based on an analysis of approximately 4,000 singleton pregnancies complicated by type 1 diabetes and over 1.8 million controls over 23 years between 1996–2018, we found a 2-4 times elevated risk of stillbirth, perinatal mortality, LGA, C-section, requirement of NICU care, and low APGAR score in type 1 diabetes that was partly explained by the fact that type 1 diabetes patients were older, delivered earlier, and had a more frequently adverse pregnancy outcomes in their medical history. The risk of congenital malformations was only increased by approximately 50% and was less affected by adjustment. The risk of SGA was significantly lower by 42% compared to controls.

Over time, the risk of stillbirth, perinatal mortality, SGA, and NICU care improved for controls, while the risk of LGA, C-section, congenital malformation, and low APGAR score increased in unadjusted models. As for pregnancies affected by type 1 diabetes, the risk of LGA, C-section, and risk of NICU care changed in the same direction as in controls, while the risk of low APGAR scores decreased over time. Adjusted rate ratios that take into account changes of population characteristics over time, showed similar changes in both populations between 1996 and 2018 with the exception of decreasing rate ratios for C-section, low APGAR scores (p<0.05), and NICU treatment (p=0.052). While the difference between cases and controls for C-sections and NICU care remained significant throughout the observation period, the risk of low APGAR scores became similar in cases and controls after 2009.

Our results for the frequency of adverse pregnancy outcomes in the background population roughly support the observations made for Hungary in other studies. In our population, the occurrence of stillbirth was 1.8% in type 1 diabetes and 0.5% in control pregnancies. According to the results of the European Perinatal Health Report 2015–2019 (EPHR), Hungary ranks 25^th^ in Europe with its 4.3 stillbirths per 1000 births (median: 3.2 per 1000 births, IQR: 2.8–3.8) (15). The occurrence of SGA was 8.2% in Hungary that was also high compared to the 4% to 11% range in Europe (15). Our findings showed an even higher rate of SGA (11.5%) that probably relates to the fact that we used a newer percentile chart on the whole population (14). Conversely, LGA affected approximately 10% of pregnancies based on a study summarizing the results of 15 European countries (16). In our study, LGA affected 11.1% of healthy pregnancies, again probably related to the use of the newest percentile tables. As for C-sections, Hungary ranks 25^th^ in Europe with its 41.5 C-sections per 1000 births (median: 26 per 1000 births IQR: 20.7–32.1) (15).

Our observations made regarding the higher risk of all investigated pregnancy outcomes in the population affected by type 1 diabetes are corroborated by other studies. Specifically, both the absolute risk of stillbirth (1.8%) and the relative risk (3.7) compared to controls well corresponds to observations from other high income countries from the early 90s to 2010s (17–25). Similarly, the observed rates and relative risk of perinatal mortality of 2.1% and 3.09 completely overlaps with reports from the literature (17–22, 24, 25). In our study population, the risk of SGA was decreased in type 1 diabetes compared to controls. Although this outcome is relatively infrequently reported, there is some support for our finding (20). In our study, LGA affected 11.1% of healthy pregnancies, while for type 1 diabetes the estimate ran as high as 29.8% translating to a relative risk of 2.71. While the literature mostly confirms the increased risk of LGA in type 1 diabetes pregnancies, both the absolute and relative risks seem to be generally higher, with absolute risks going as high as 63% and RRs up to 11.5 (6, 20, 21, 25, 26). The risk of C-section was doubled in type 1 diabetes compared to controls in our study that is line with other observations of increased risks from other countries. However, the RRs are not easy to compare, given the wide range of C-sections in the general population across high income countries (6, 21, 26–28). Similarly, the risk of NICU admission is increased in type 1 diabetes according to both our observations and the literature (6, 28). In general, the rate of congenital anomalies was found to be elevated in type 1 diabetes pregnancies compared to controls both in our study and in previous observations. However, direct comparisons with the literature are hindered by the fact that we excluded deliveries terminating before 24 weeks of gestation leading to lower absolute and relative risks compared to the literature (17, 19, 21, 24, 28). Median APGAR scores are lower, as well as the risk of a low APGAR score is increased in type 1 diabetes compared to controls confirming our observations (27–29).

Major characteristics of our study population changed substantially from 1996 to 2018. The age of mothers with and without type 1 diabetes increased similarly by ~3 years. Similar observations are available for other high-income countries (15, 30–33). At the same time, we observed decreasing parity in both type 1 diabetes and controls, similarly to other European countries (15, 30). Furthermore, a decreasing gestational age at delivery seems to be universal finding in our and other studies (10, 15, 34). The increasing age of pregnant women is probably the driver of the more frequent occurrence of primary hypertension. Furthermore, measures of obesity (although we do not have this measure in our database) are also showing increasing trends both in people with and without type 1 diabetes concurrent with our study period in Hungary and elsewhere in Europe that may also increase the risk of adverse pregnancy outcomes and concurrent diseases (32, 35). As type 1 diabetes patients and controls have different baseline characteristics, and these characteristics may change differently in cases and controls, it is extremely important to eliminate their effect when examining temporal changes in comparative risks between cases and controls.

Among healthy pregnancies, we observed beneficial decreasing trends in the rates of stillbirth, perinatal mortality, SGA, and the risk of NICU care that well corresponds to European and worldwide trends (15, 36). In contrast, rates of C-section, low APGAR score, and congenital malformations increased over time. While the frequency of C-sections and its temporal changes shows high heterogeneity within Europe, the increasing trend in Hungary seems to be continuing also for the last 5 years (10, 15). Although these increases could be partially linked to the increasing age, BMI, and consequently higher risk of comorbidities of the mothers, changes in C-sections may also be explained by a more frequent choice of C-sections over natural birth by either patients or physicians.

The trends of pregnancy outcomes showed a somewhat different picture for pregnancies affected by type 1 diabetes compared to controls. We found no significant changes in the risk of stillbirth, perinatal mortality, and congenital malformation. While these findings could partly be related to the limited power of our analysis for these relatively rare outcomes, it is notable that stillbirth and perinatal mortality remained stubbornly persistent in the UK and Canada between the mid-nineties and early 2000s, probably partly explained by the decreasing participation in antenatal care of participants (37, 38). Another study found an initial increase followed by a decrease in stillbirth risk in Norway from 1985 to 1998 that was related to improving diabetes management of type 1 diabetes (22). The prevention of congenital malformations requires good glycemic control in the early pregnancy period that highlights the importance of pre-pregnancy counselling and care (25). There is some evidence in the literature of decreasing trends of congenital malformations, however the observed trends did not exceed those in the background population (38–40). While the risk of LGA increased parallel to control pregnancies, the literature is equivocal on this outcome. While two studies reported increasing rates of LGA (21, 41), another study found no change despite improving protocols and medication regimens between the nineties and early 2000s (42). To decrease macrosomia, proper glycemic control seems to be important especially in the first trimester along with proper weight control of participants (42, 43). We found somewhat improving rates of SGA, NICU care, and low APGAR scores in type 1 diabetes pregnancies that well correspond to the findings of a Polish tertiary care center (41).

While the above results show overall changes in the actual number of events, they provide little information on how the risk would change over time if the population would remain stable. Our analyses on the trends of rate ratios of the outcomes adjusted for confounders help us answer these questions. We found that the rate ratios between type 1 diabetes and controls decreased for three of the outcomes (C-section, NICU care, and low APGAR score), while for the rest, the changes were similar in cases and controls. The decrease in the occurrence of low APGAR scores was not only more pronounced in type 1 diabetes, but it decreased to an extent that it was no longer significantly different between cases and controls. For C-section rates that increased both in cases and controls, the increase was less pronounced in type 1 diabetes. Similarly, the decrease in NICU care was faster in cases compared to controls. These beneficial changes may reflect an improvement in overall pregnancy care protocols, glycemic control, and delivery procedures (41). It should be noted that these outcomes are less strongly related to early glycemic control compared to congenital malformations. Proper glycemic control has been linked to decreasing rates of other negative pregnancy outcomes: every 1% reduction of HbA1c is associated with an approximately 50% reduction in the risk of unwanted pregnancy outcomes (25). However HbA1c alone is not necessarily the only measure that should be used to monitor therapeutic goals, as mothers with normal HbA1c levels may still have a higher risk of adverse pregnancy outcomes (25). To approximate the pregnancy outcomes of the population affected by diabetes to that of healthy pregnancies, in addition to optimal glycemic control, participation in antenatal care, and administration of supplements, such as folic acid, should also be implemented as part of a multidisciplinary care program (25).

Our study has limitations that have to be acknowledged. Even though the included population is very large, for some rare outcomes, our trend analyses have a limited statistical power and thus important differences may remain unobserved. As we used registry data, misdiagnosis and misclassification of outcomes and predictors is a possibility. It should be noted, however that the Tauffer registry is not used for reimbursement purposes and thus selective misclassification (an important source of bias) is unlikely. Furthermore, we had no information on potentially important confounders, such as smoking, social status, measures of obesity. Given that we used anonymized data, we could not adjust for the multilevel structure of the data, although the use of bootstrapping and robust confidence intervals gives us some support that our findings are valid. Due to the lack of information on glycemic control, blood pressure, and other biological variables, we could not investigate causal biological factors behind the observed changes.

Major strengths of our study include its large sample size and long follow-up. Actually, ours is one of the longest studies investigating trends in pregnancy outcomes in type 1 diabetes compared to the background population. Given that most variables in our analysis are mandatory fields in the database, we could include almost all singleton births in Hungary between 1996 and 2018. Moreover, our main and sensitivity analyses showed similar findings that further confirm the robustness of our observations. Furthermore, the investigation of nulliparas allowed us to remove the multilevel structure of the data.

## Conclusion

In conclusion, although we found that the rates of some outcomes (such as SGA, NICU care, and low APGAR score) improved in pregnancies complicated by type 1 diabetes, the risk of LGA and C-sections increased during the over 20-year observation period. Participating countries of the St Vincent Declaration, unanimously agreed to approximate pregnancy health outcomes of pregnancies complicated by type 1 diabetes to that of healthy pregnancies in 1989. However, we only found that this target was achieved for the occurrence of low APGAR scores. Furthermore, we found decreasing differences in terms of C-sections and NICU care but despite these beneficial trends, significant differences remained between type 1 diabetes cases and controls. To achieve all targets of the St Vincent Declaration, further improvements are required in pre-pregnancy and pregnancy management and care of women with type 1 diabetes.

## Data availability statement

The original contributions presented in the study are included in the article/**Supplementary Material**. Further inquiries can be directed to the corresponding author.

## Author contributions

AT conceptualized and designed the study. Analysis and interpretation were handled by all authors. AT, VF-P, and MS wrote the first draft of the manuscript. All authors took critical part in the revision process. All authors provide approval for publication of the content and agree to be accountable for all aspects of the work in ensuring that questions related to the accuracy or integrity of any part of the work are appropriately investigated and resolved.
