# Supplementary material for: Trend of pregnancy outcomes in type 1 diabetes compared to control women: a register-based analysis in 1996-2018
Source: Front Endocrinol (Lausanne). 2023 Jul 12;14:1232618. doi: 10.3389/fendo.2023.1232618 (PMC10369353; doi:10.3389/fendo.2023.1232618)
Supplement: Supplementary file 1 [file Table_1.docx]

| **Supplement Table 1** Rate ratios for different pregnancy outcomes based on Poisson regression with hierarchical adjustment for potential confounders in singleton nulliparas affected by type 1 diabetes compared to controls over 3 time periods (1996–2002, 2003–2010, 2011–2018) | | | | | | |
| --- | --- | --- | --- | --- | --- | --- |
|  |  | Type 1 diabetes | Controls | Unadjusted | Model 1† | Model 2†† |
|  |  | n=2,110 | n=901,124 |  |  |  |
|  | Time period | n (%) | n (%) | RR (95% CI) | RR (95% CI) | RR (95% CI) |
| n | 1996–2002 | 415 (19.7) | 218,022 (24.2) | – | – | – |
|  | 2003–2010 | 739 (35.0) | 341,746 (37.9) | – | – | – |
|  | 2011–2018 | 956 (45.3) | 341,356 (37.9) | – | – | – |
| Stillbirth | 1996–2002 | 7 (1.70) | 1,050 (0.50) | 3.50 (1.68–7.32)* | 2.05 (0.99–4.22) | 2.07 (1.00–4.27)* |
|  | 2003–2010 | 11 (1.50) | 1,457 (0.40) | 3.50 (1.94–6.29)* | 1.53 (0.86–2.73) | 1.55 (0.87–2.75) |
|  | 2011–2018 | 11 (1.20) | 1,323 (0.40) | 2.97 (1.65–5.36)* | 1.49 (0.83–2.67) | 1.50 (0.84–2.68) |
|  | P for heterogeneity** | 0.699 | <0.001 | 0.912 | 0.773 | 0.768 |
|  | P for trend** | 0.402 | <0.001 | 0.703 | 0.548 | 0.540 |
| Perinatal mortality | 1996–2002 | 8 (1.90) | 1,642 (0.80) | 2.56 (1.29–5.09)* | 1.67 (0.84–3.30) | 2.21 (1.10–4.43)* |
|  | 2003–2010 | 16 (2.20) | 2,045 (0.60) | 3.63 (2.23–5.90)* | 1.63 (1.07–2.47) | 1.83 (1.18–2.83)* |
|  | 2011–2018 | 14 (1.50) | 1,709 (0.50) | 2.93 (1.74–4.93)* | 1.52 (0.93–2.50) | 1.72 (1.01–2.94)* |
|  | P for heterogeneity** | 0.551 | <0.001 | 0.689 | 0.970 | 0.851 |
|  | P for trend** | 0.424 | <0.001 | 0.840 | 0.815 | 0.600 |
| Small for gestational age | 1996–2002 | 33 (8.00) | 30,391 (13.9) | 0.57 (0.41–0.79)* | 0.65 (0.46–0.90)* | 0.64 (0.46–0.90)* |
|  | 2003–2010 | 67 (9.10) | 41,139 (12.0) | 0.75 (0.60–0.95)* | 0.84 (0.67–1.06) | 0.84 (0.67–1.05) |
|  | 2011–2018 | 75 (7.80) | 40,859 (12.0) | 0.66 (0.53–0.82)* | 0.73 (0.59–0.91)* | 0.72 (0.58–0.90)* |
|  | P for heterogeneity** | 0.639 | <0.001 | 0.369 | 0.401 | 0.400 |
|  | P for trend** | 0.767 | <0.001 | 0.632 | 0.711 | 0.735 |
| Large for gestational age | 1996–2002 | 93 (22.4) | 17,811 (8.20) | 2.74 (2.29–3.28)* | 2.45 (2.05–2.94)* | 2.43 (2.03–2.92)* |
|  | 2003–2010 | 204 (27.6) | 32,465 (9.50) | 2.90 (2.58–3.26)* | 2.61 (2.32–2.94)* | 2.59 (2.30–2.91)* |
|  | 2011–2018 | 253 (26.5) | 30,186 (8.80) | 2.99 (2.69–3.33)* | 2.70 (2.43–3.01)* | 2.67 (2.40–2.97)* |
|  | P for heterogeneity** | 0.146 | <0.001 | 0.711 | 0.656 | 0.679 |
|  | P for trend** | 0.218 | <0.001 | 0.509 | 0.428 | 0.45 |
| Abbreviations: 95% CI: 95% Confidence Interval; RR: Rate Ratio. | | | | | | |
| *p<0.05 | | | | | | |
| **p-values for heterogeneity and linear trend of rate ratios were computed with Poisson regression. | | | | | | |
| †Model 1 was adjusted for gestational age, maternal age, and sex of infant. | | | | | | |
| ††Model 2 was adjusted for covariates of Model 1 and presence of prior adverse pregnancy outcome, prior livebirth, and pre-pregnancy hypertension. | | | | | | |

| **Supplement Table 2.** Rate ratios for different pregnancy outcomes based on Poisson regression with hierarchical adjustment for potential confounders in singleton nulliparas affected by type 1 diabetes compared to controls over 3 time periods (1996–2002, 2003–2010, 2011–2018) | | | | | | |
| --- | --- | --- | --- | --- | --- | --- |
|  |  | Type 1 diabetes | Controls | Unadjusted | Model 1† | Model†† |
|  |  | n=2,110 | n=901,124 |  |  |  |
|  | Time period | n (%) | n (%) | RR (95% CI) | RR (95% CI) | RR (95% CI) |
| n | 1996–2002 | 415 (19.7) | 218,022 (24.2) | – | – | – |
|  | 2003–2010 | 739 (35.0) | 341,746 (37.9) | – | – | – |
|  | 2011–2018 | 956 (45.3) | 341,356 (37.9) | – | – | – |
| Caesar section | 1996–2002 | 238 (57.3) | 50,494 (23.3) | 2.48 (2.28–2.69)* | 2.24 (2.05–2.44)* | 2.20 (2.02–2.40)* |
|  | 2003–2010 | 483 (65.4) | 110,076 (32.2) | 2.03 (1.93–2.14)* | 1.84 (1.74–1.94)* | 1.81 (1.72–1.91)* |
|  | 2011–2018 | 657 (68.7) | 136,185 (39.9) | 1.72 (1.65–1.80)* | 1.54 (1.47–1.61)* | 1.50 (1.43–1.57)* |
|  | P for heterogeneity** | <0.001 | <0.001 | <0.001 | <0.001 | <0.001 |
|  | P for trend** | <0.001 | <0.001 | <0.001 | <0.001 | <0.001 |
| NICU | 1996–2002 | 91 (22.0) | 13,206 (6.10) | 3.63 (3.02–4.35)* | 2.16 (1.78–2.64)* | 2.13 (1.75–2.609* |
|  | 2003–2010 | 180 (24.4) | 19,113 (5.60) | 4.37 (3.84–4.96)* | 2.35 (2.04–2.71)* | 2.32 (2.01–2.67)* |
|  | 2011–2018 | 181 (18.9) | 20,107 (5.90) | 3.22 (2.82–3.67)* | 1.84 (1.62–2.10)* | 1.82 (1.60–2.07)* |
|  | P for heterogeneity** | 0.025 | <0.001 | 0.004 | 0.038 | 0.040 |
|  | P for trend** | 0.069 | 0.108 | 0.080 | 0.064 | 0.064 |
| Congenital malformations | 1996–2002 | 14 (3.40) | 3,755 (1.70) | 1.96 (1.17–3.28)* | 1.88 (1.12–3.15)* | 1.85 (1.10–3.10)* |
|  | 2003–2010 | 23 (3.10) | 6,277 (1.80) | 1.70 (1.14–2.55)* | 1.62 (1.08–2.43)* | 1.60 (1.07–2.39)* |
|  | 2011–2018 | 27 (2.80) | 6,114 (1.80) | 1.58 (1.09–2.29)* | 1.54 (1.06–2.24)* | 1.51 (1.04–2.19)* |
|  | P for heterogeneity** | 0.852 | 0.007 | 0.800 | 0.829 | 0.819 |
|  | P for trend** | 0.571 | 0.127 | 0.506 | 0.558 | 0.541 |
| APGAR Score ≤6 | 1996–2002 | 5 (1.20) | 1,754 (0.80) | 1.50 (0.63–3.58) | 1.25 (0.54–2.89) | 1.25 (0.54–2.90) |
|  | 2003–2010 | 11 (1.50) | 1,930 (0.60) | 2.65 (1.47–4.77)* | 1.61 (0.92–2.82) | 1.61 (0.92–2.82) |
|  | 2011–2018 | 10 (1.00) | 8,028 (2.40) | 1.39 (0.73–2.67) | 0.98 (0.52–1.85) | 0.98 (0.52–1.85) |
|  | P for heterogeneity** | 0.793 | <0.001 | 0.305 | 0.514 | 0.513 |
|  | P for trend** | 0.777 | 0.461 | 0.716 | 0.552 | 0.551 |
| Abbreviations: APGAR Score: Appearance, Pulse, Grimace, Activity and Respiration Score; 95% CI: 95% Confidence Interval; NICU: Neonatal Intensive Care Unit; RR: Rate Ratio. | | | | | | |
| *p<0.05 | | | | | | |
| **p-values for heterogeneity and linear trend of rate ratios were computed with Poisson regression. | | | | | | |
| †Model 1 was adjusted for gestational age, maternal age, and sex of infant. | | | | | | |
| ††Model 2 was adjusted for covariates of Model 1 and presence of prior adverse pregnancy outcome, prior livebirth, and pre-pregnancy hypertension. | | | | | | |
| Abbreviations: APGAR Score: Appearance, Pulse, Grimace, Activity and Respiration Score; 95% CI: 95% Confidence Interval; NICU: Neonatal Intensive Care Unit; RR: Rate Ratio. | | | | | | |
